# Alcohol Activates TGF-Beta but Inhibits BMP Receptor-Mediated Smad Signaling and Smad4 Binding to Hepcidin Promoter in the Liver

**DOI:** 10.1155/2012/459278

**Published:** 2011-10-23

**Authors:** Lisa Nicole Gerjevic, Na Liu, Sizhao Lu, Duygu Dee Harrison-Findik

**Affiliations:** Division of Gastroenterology and Hepatology, Department of Internal Medicine, University of Nebraska Medical Center, 95820 UNMC, Omaha, NE 68198-5820, USA

## Abstract

Hepcidin, a key regulator of iron metabolism, is activated by bone morphogenetic proteins (BMPs). Mice pair-fed with regular and ethanol-containing L. De Carli diets were employed to study the effect of alcohol on BMP signaling and hepcidin transcription in the liver. Alcohol induced steatosis and TGF-beta expression. Liver BMP2, but not BMP4 or BMP6, expression was significantly elevated. Despite increased BMP expression, the BMP receptor, and transcription factors, Smad1 and Smad5, were not activated. In contrast, alcohol stimulated Smad2 phosphorylation. However, Smad4 DNA-binding activity and the binding of Smad4 to hepcidin promoter were attenuated. In summary, alcohol stimulates TGF-beta and BMP2 expression, and Smad2 phosphorylation but inhibits BMP receptor, and Smad1 and Smad5 activation. Smad signaling pathway in the liver may therefore be involved in the regulation of hepcidin transcription and iron metabolism by alcohol. These findings may help to further understand the mechanisms of alcohol and iron-induced liver injury.

## 1. Introduction

Alcoholic liver-disease patients frequently display evidence of iron overload [1–5]. Alcohol-induced iron overload enhances the production of free radicals and proinflammatory cytokines [6, 7]. However, the underlying mechanisms of iron accumulation observed in alcoholic liver disease are unclear. We and others have recently shown a role for hepcidin in alcohol-induced increases in iron transport [8–13]. Hepcidin is a circulatory antimicrobial peptide synthesized by the liver [14, 15]. It plays a pivotal role in iron homeostasis by inhibiting iron uptake in the duodenum and iron export in reticuloendothelial macrophages [16, 17]. Alcohol downregulates hepcidin expression in the liver, which leads to an increase in duodenal iron transporter expression [9]. However, how alcohol suppresses hepcidin transcription in the liver is still unclear. 

Bone morphogenetic proteins (BMPs) belong to the transforming growth factor beta (TGF-*β*), superfamily of growth factors [18]. BMP2, BMP4, BMP6 and BMP9 have all been reported to regulate hepcidin transcription [19–22]. However, transgenic mouse studies have recently suggested that BMP6, is involved in the regulation of hepcidin expression *in vivo* [23, 24]. Moreover, iron has been shown to induce BMP6 mRNA expression and Smad5 phosphorylation [25–27]. Similar to TGF-*β* receptor, the binding of BMP ligands to type I and type II BMP receptor serine/threonine kinases leads to the phosphorylation and activation of type I BMP receptor (BMPR-I) [28]. Activated BMPR-I in turn phosphorylates the receptor-regulated Smad (R-Smad) family of transcription factors: Smad1, Smad5, and Smad8 [29]. On the other hand, activated TGF-*β* receptor induces the phosphorylation of Smad2 and Smad3. Upon phosphorylation, these R-Smads form a complex with the common mediator of Smad signaling, Smad4. The Smad complexes subsequently translocate into the nucleus where they participate in the regulation of gene transcription [30, 31]. Of note, liver-specific disruption of Smad4 leads to a decrease in hepcidin expression and accumulation of iron in liver, kidney, and pancreas [32]. 

The involvement of the profibrogenic cytokine, TGF-*β* in alcohol-induced liver injury has been well-established [33, 34]. However, the role of BMPs and BMP receptor-mediated signaling in alcoholic liver disease is largely unknown. In this study, we examine the effect of alcohol on BMP expression and BMP receptor-mediated regulation of hepcidin transcription in the liver *in vivo*. Alcohol and iron play a synergistic role in the pathogenesis of alcoholic liver disease. These studies will help us to further understand the mechanisms of liver injury induced by iron and alcohol.

## 2. Materials and Methods

### 2.1. Animal Experiments

Animal experiments were approved by the Animal Ethics Committee at the University of Nebraska Medical Center. C57BL/6 NCR male mice (NIH) were housed individually and pair-fed with either regular or ethanol-containing Lieber De Carli liquid diets (Dyets, Inc., cat no: 710027, 710260, resp.), as described previously [12]. The ethanol content of the diet was gradually increased over a 9-day period to 5% (no ethanol for 3 days, 1% for 2 days, 2% for 2 days, and 3% for 2 days). Mice were exposed to 5% ethanol for 4 weeks. For iron experiments, mice were fed initially with a custom prepared egg-white-based solid rodent diet [35] containing 0.02% carbonyl iron (F614, Bio-Serv, Inc.) for one week to achieve a basal hepcidin expression level. Subsequently, they were fed with 0.2% or 2% carbonyl iron diets for 3 weeks to achieve normal and iron overload states, respectively, as published previously [12].

### 2.2. RNA Isolation, cDNA Synthesis, and Real-Time Quantitative PCR Analysis

RNA isolation, cDNA synthesis, and quantitative PCR were performed, as published previously [9]. The sequences of Taqman fluorescent probe (5^'^ 6-[FAM]; 3^'^ [TAMRA-Q]) and primers are shown in Table 1.

### 2.3. Western Blotting, Immunoprecipitation, and Immunohistochemistry

Total liver cell lysates were prepared by homogenizing mouse livers in lysis buffer [10 mM Tris/HCl (pH 7.4), 100 mM NaCl, 5 mM EDTA, 10% glycerol, 1 mM PMSF, complete protease inhibitor cocktail (Roche Diagnostics Corp.), phosphatase inhibitor cocktail A (Santa Cruz, sc-45044), and 1% Triton-X-100]. The lysates were subsequently incubated on ice for 20 min. and centrifuged (3000x g) for 5 min. at 4°C. Supernatants were employed for western blot or immunoprecipitation experiments. Western blots were performed, as described previously [12, 36]. Anti-phospho-Smad2, anti-phosho-Smad1/5, anti-Smad2, and anti-Smad5 antibodies were obtained commercially (cell signaling). For immunoprecipitations, 500 *μ*g of liver lysate protein was incubated with BMPR-I antibody or normal rabbit IgG (Santa Cruz) and protein A/G PLUS-Agarose preblocked with BSA (Santa Cruz). Immunocomplexes eluted by nonreducing SDS buffer were resolved on 10% polyacrylamide gels and immunoblotted with anti-phosphoserine (Millipore) or BMPR-I antibodies (Santa Cruz). Alkaline phosphatase-conjugated anti-mouse (Millipore) or anti-rabbit (SouthernBiotech) light chain-specific immunoglobulins were used as secondary antibodies. Immunostaining of paraffin embedded liver sections with TGF-*β* (Abcam) or BMP2 (Santo Cruz) antibodies were performed by Vectastain ABC kit (Vector Labs), according to manufacturer's instructions.

### 2.4. Electrophoretic Mobility Gelshift Assay (EMSA)

Mouse liver nuclear lysate isolation and EMSA were performed, as described [9]. Briefly, the consensus and mutant Smad4 oligonucleotides (Santa Cruz) were labeled by T4 polynucleotide kinase and ^32^P-*γ*-ATP (Perkin Elmer, 3.000 Ci/moL, 10 mCi/mL). 7 *μ*g of nuclear extract protein and 100.000 cpm of ^32^P-labeled Smad probes were used for each binding reaction. Protein and DNA complexes were resolved on 7% nondenaturing polyacrylamide gels and radiolabeled bands were visualized by autoradiography. For competition assays, unlabeled consensus Smad oligonucleotide in 30-fold excess was incubated with nuclear lysates on ice prior to the addition of the ^32^P-labeled consensus Smad probe.

### 2.5. Chromatin Immunoprecipitation (CHIP)

CHIP was performed, as described [37]. Chromatin isolated from formalin-fixed mouse liver was sheared by sonication and immunoprecipitated by using control IgG (cell signaling) or anti-Smad4 antibody (cell signaling) and protein A/G beads (Santa Cruz). An aliquot of precleared chromatin was saved as total input DNA prior to the immunoprecipitation. Coimmunoprecipitated DNA and total input DNA were analyzed by PCR using primers (forward 5^'^-gccatactgaaggcactga^'^3; reverse 5^'^-gtgtggtggctgtctagg-3^'^) specific for mouse hepcidin promoter.

### 2.6. Statistical Analysis

Statistical analysis of differences in treatment groups was performed by using the nonparametric Mann-Whitney test and Student's *t*-test.

## 3. Results

**Figure 1:**
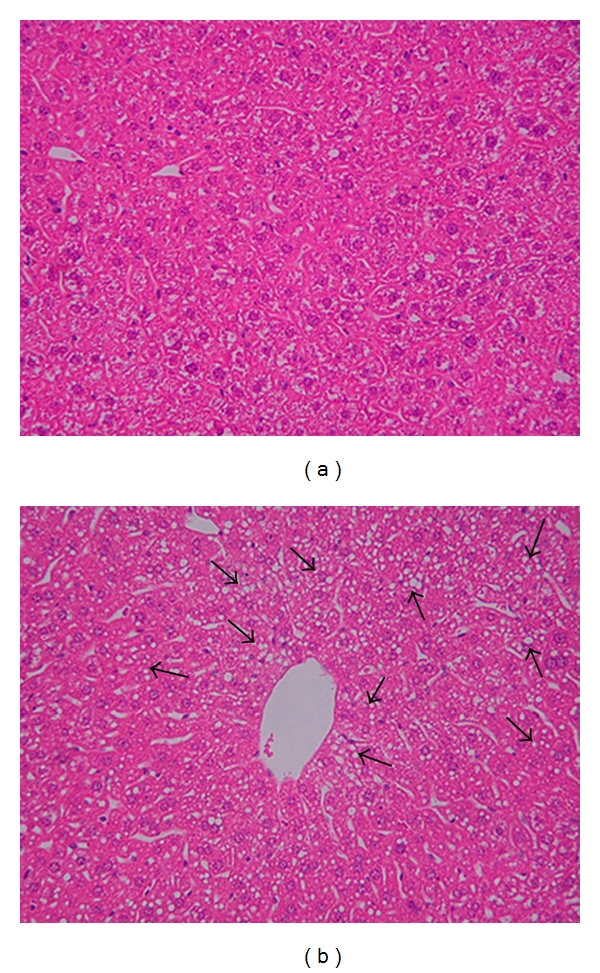
*Liver Histology*. The fixed liver sections of mice fed with regular (a) or ethanol-containing (b) Lieber-De Carli liquid diets were stained with hematoxylin and eosin (a, b), as described in Materials and Methods (original magnification 20X). The arrows indicate steatosis (b).

**Figure 2:**
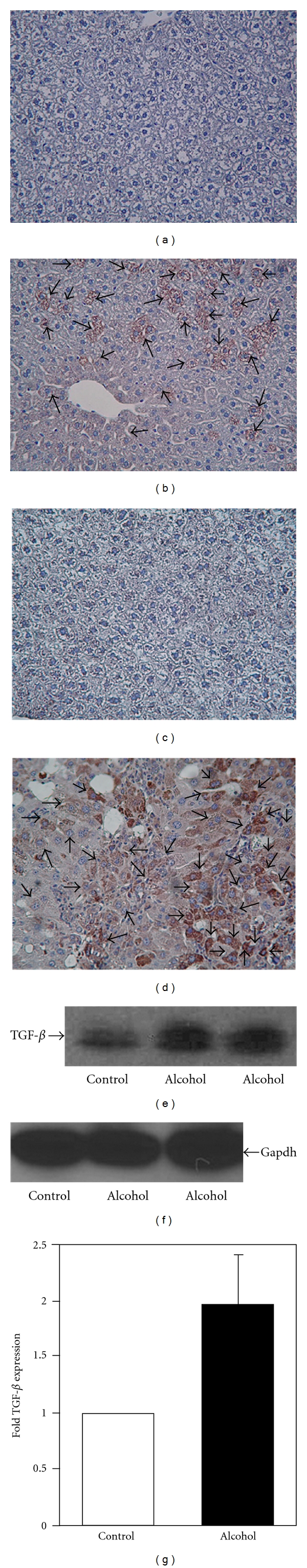
*TGF-*β* Expression*. The fixed liver sections of mice fed with regular (a) or ethanol-containing (b) Lieber-De Carli liquid diets were immunostained with an anti-TGF-*β* antibody, as described in Materials and Methods. Liver sections from mice injected (i.p.) with sunflower oil, as control (c) or carbon tetrachloride (d) for 8 weeks were also immunostained with the anti-TGF-*β* antibody to serve as positive controls for TGF-*β* staining. The arrows indicate TGF-*β* expression (b, d) (original magnification 20X). Whole cell lysates isolated from the livers of mice fed with regular (control) or ethanol-containing (alcohol) Lieber-De Carli liquid diets were employed to determine TGF-*β* protein expression by western blotting, as described in Materials and Methods (e). An anti-gapdh antibody was employed to confirm equal protein loading (f). TGF-*β* protein expression, normalized to gapdh, in alcohol-treated mice was expressed as fold expression of that in control mice and was quantified by scanning autoradiographs by a densitometer (g).

**Figure 3:**
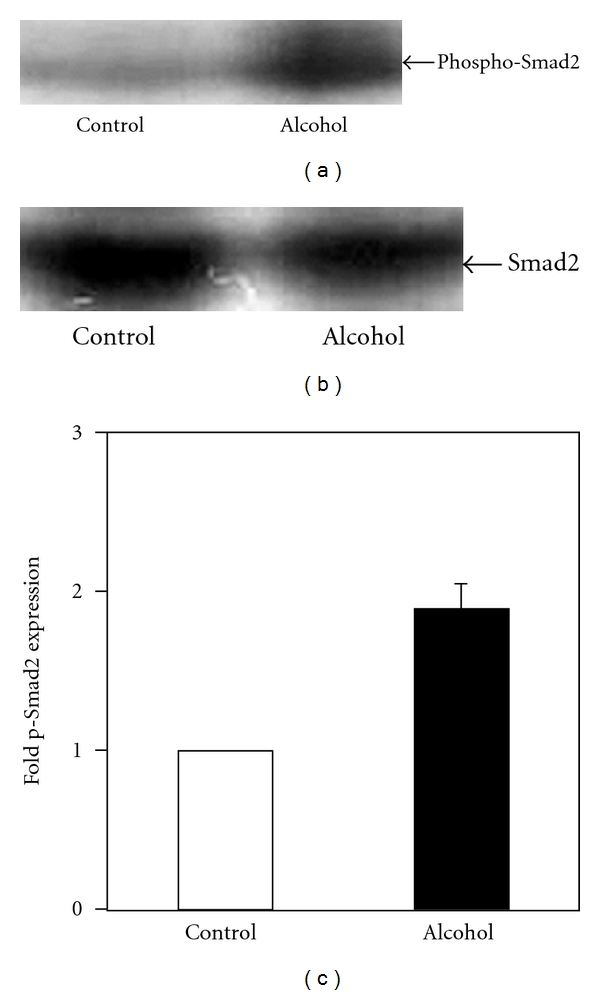
*Smad2 Activation.* The phosphorylation of Smad2 protein in the liver lysates of mice fed with regular (control) or ethanol-containing (alcohol) Lieber-De Carli liquid diets was determined by western blotting employing an anti-phospho-Smad2 antibody, as described in Materials and Methods. (b) An antibody recognizing the total levels of Smad2 protein was employed as a control to confirm equal protein loading. (c) Autoradiographs from different experiments (*n* = 3) were scanned by a densitometer, and phospho-Smad2 (p-Smad2) expression in each sample was quantified by normalizing to total Smad2 protein expression. Normalized phospho-Smad2 expression in alcohol-treated mice was expressed as fold expression of that in control mice.

**Figure 4:**
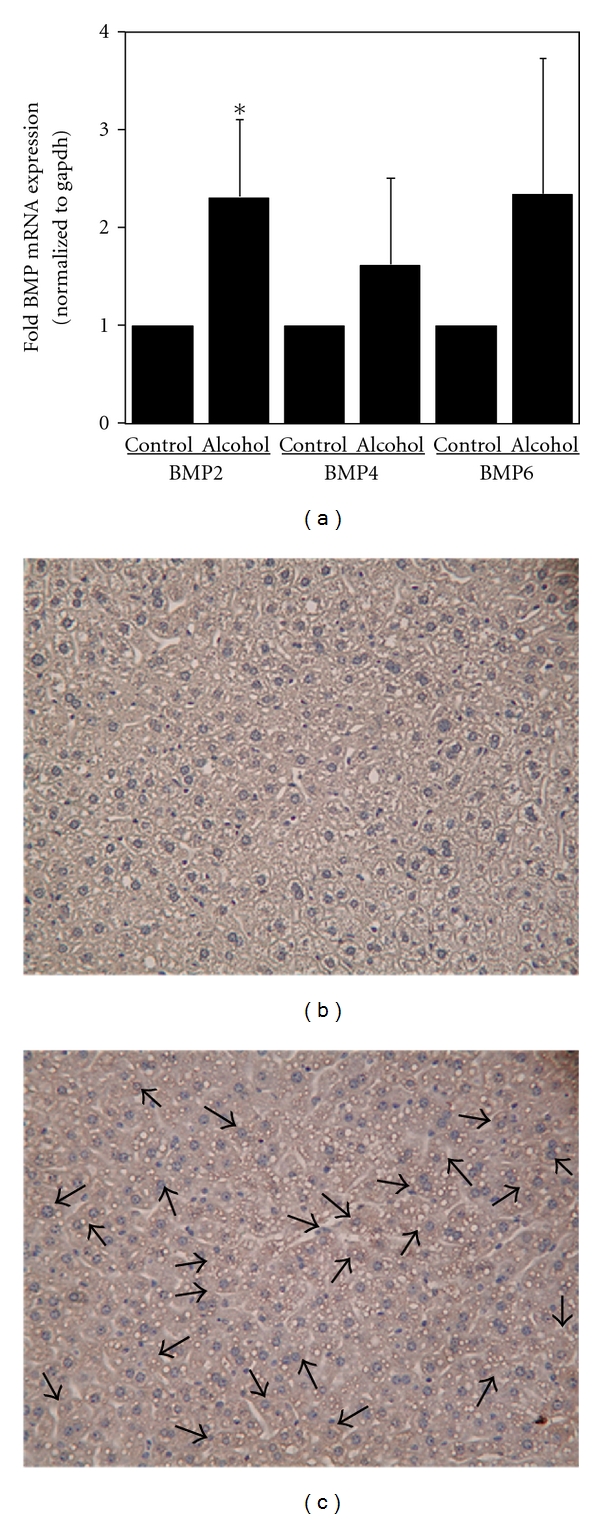
*Alcohol and BMP2, BMP4, BMP6 Expression*. (a) cDNA was synthesized from liver RNA of mice fed with regular (control) or ethanol-containing (alcohol) Lieber-De Carli liquid diets, as described in Materials and Methods. It was employed in real-time PCR to detect bone morphogenetic protein (BMP) expression. The mRNA expression in alcohol-fed mice was expressed as fold of that in pair-fed control mice fed with regular diet (mean ± S.E.M.; *n* = 3, 4 mice per group). Asterisks indicate statistical significance (*P* < 0.05). The fixed liver sections of mice fed with regular (b) or ethanol-containing (c) Lieber-De Carli liquid diets were immunostained with an anti-BMP2 antibody, as described in Materials and Methods. The arrows indicate BMP2 expression (original magnification 20X).

**Figure 5:**
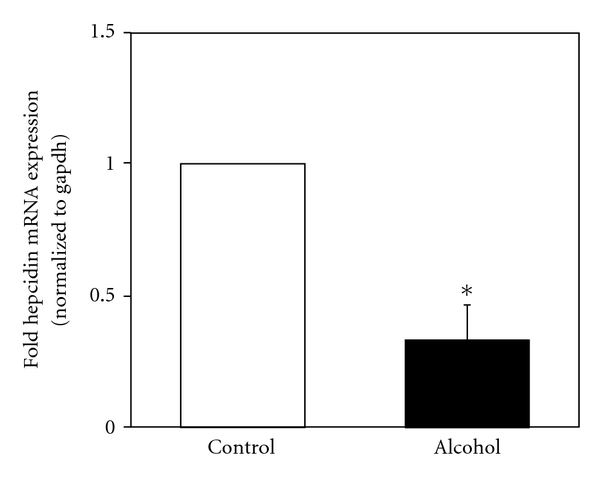
*Alcohol and Hepcidin Expression*. cDNA was synthesized from liver RNA of mice fed with regular (control) or ethanol-containing (alcohol) Lieber-De Carli liquid diets, as described in Materials and Methods. It was employed in real-time PCR to detect hepcidin expression. The mRNA expression in alcohol-fed mice was expressed as-fold of that in pair-fed control mice fed with regular diet (mean ± S.E.M.; *n* = 3, 4 mice per group). Asterisks indicate statistical significance (*P* < 0.05).

In order to study the effect of chronic alcohol consumption on the expression of different bone morphogenetic proteins (BMPs) and signaling in the liver, we employed wild-type mice pair-fed with regular (control) or ethanol-containing Lieber De Carli diets, as described in Materials and Methods. Mice fed with alcohol for 4 weeks displayed significant lipid accumulation in the liver, compared to control mice fed with regular L. De Carli diet, as shown by hematoxylin and eosin staining (Figures 1(a) and 1(b)). Similarly, chronic alcohol consumption resulted in increased transforming growth factor beta (TGF-*β*) expression in the liver, as shown by immunostaining and western blotting (Figure 2). TGF-*β* is known to induce the phosphorylation and activation of the transcription factor, Smad2 [29]. Accordingly, western blot analysis indicated a significant twofold increase in the level of phospho-Smad2 protein expression in the livers of alcohol-fed mice compared to control mice (Figures 3(a) and 3(c)). The level of total Smad2 protein expression in the liver was not altered by alcohol (Figure 3(b)). BMPs also belong to the TGF-*β* superfamily of growth factors and activate the Smad signaling pathway [18]. However, the effect of alcohol on BMP expression is unknown. Compared to control mice, mice with chronic alcohol exposure displayed an increase in BMP2, BMP4, and BMP6 mRNA expression in the liver (Figure 4(a)). However, the median response differences in BMP4 and BMP6 expression between alcohol-fed and control mice were not statistically significant (*P* > 0.05) (Figure 4(a)). In contrast, the alcohol-induced increase in BMP2 mRNA expression in the liver was statistically significant (*P* < 0.05) (Figure 4(a)). The livers of alcohol-treated mice also exhibited an increase in BMP2 protein expression compared to control mice (Figures 4(b) and 4(c)). Mice with chronic alcohol exposure displayed a significant (*P* < 0.05) decrease in hepcidin mRNA expression in the liver (Figure 5). 

**Figure 6:**
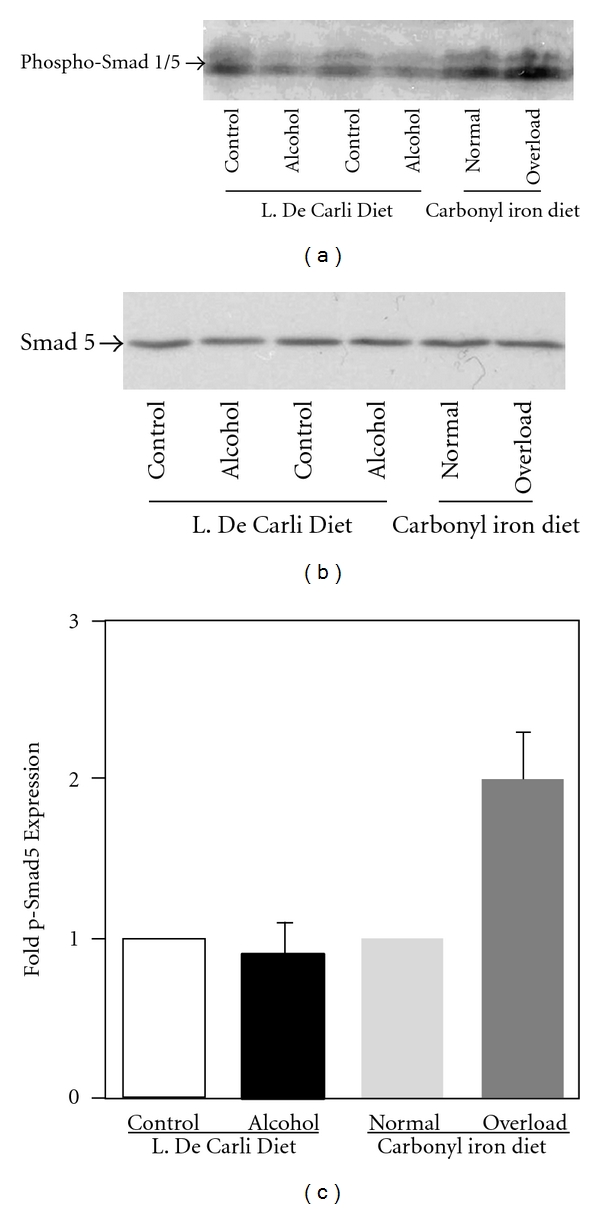
*Activation of Smad1 and Smad5*. (a) The phosphorylation of Smad1 and Smad5 proteins in the liver lysates of mice fed with regular (control) or ethanol-containing (alcohol) Lieber-De Carli liquid diets, and of mice fed with diets containing 0.2% (normal) or 2% (overload) carbonyl iron was determined by western blotting employing an anti-phospho-Smad1/5 antibody, as described in Materials and Methods. (b) An antibody recognizing the total levels of Smad5 protein was employed as a control to confirm equal protein loading. (c) Autoradiographs from different experiments (*n* = 3) were scanned by a densitometer, and phospho-Smad1/5 (p-Smad5) expression in each sample was quantified by normalizing to total Smad5 protein expression. Normalized phospho-Smad expression in alcohol or iron-fed mice was expressed as fold expression of that in the control mice.

Bone morphogenetic proteins induce intracellular signaling via the phosphorylation of the transcription factors, Smad1, Smad5, and Smad8. We performed western blots by using an antibody which recognizes both Smad1 and Smad5 phosphorylated on serine residues, as described in Materials and Methods. Unlike Smad 2 (see above), our western blot analysis did not detect a significant change in the phosphorylation of Smad1 and Smad5 proteins in the livers of alcohol-treated mice, compared to the controls (Figures 6(a) and 6(c)). Since iron has been reported to induce BMP signaling and Smad5 phosphorylation [25, 27], the livers of mice fed with iron diets (see Materials and Methods) were employed as internal controls. Accordingly, our western blot analysis detected a significant increase in Smad1 and Smad5 phosphorylation in the livers of mice with iron overload, compared to control mice with a normal iron state (Figures 6(a) and 6(c)). The level of total Smad5 protein expression in the liver was not altered by alcohol or iron treatments (Figure 6(b)). 

**Figure 7:**
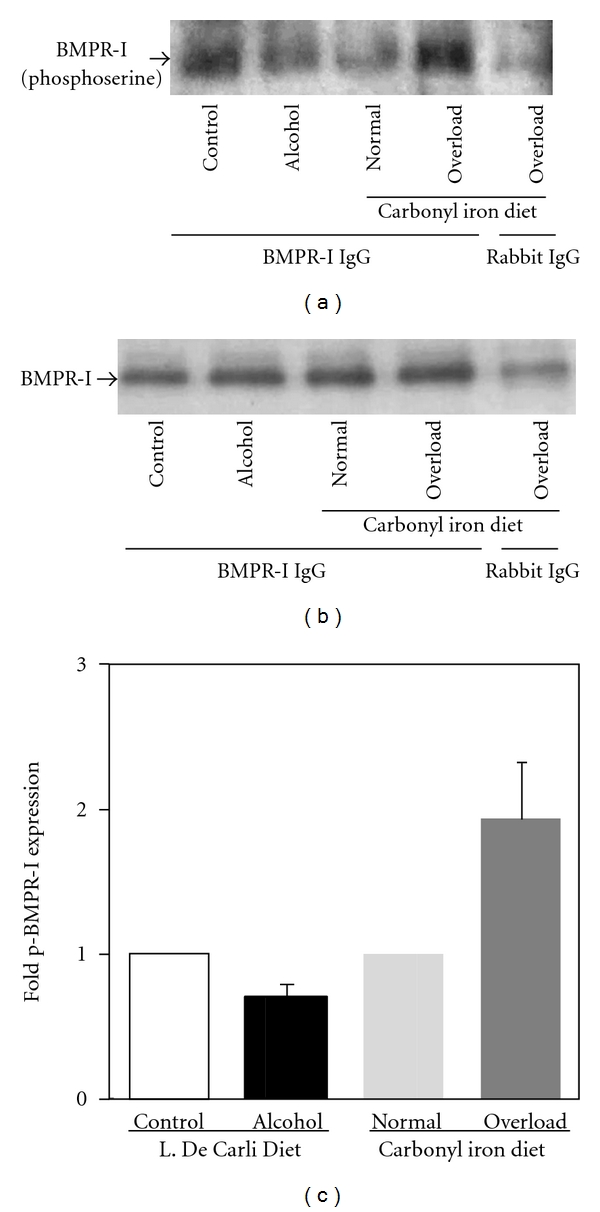
*Activation of BMPR-I*. Bone morphogenetic protein receptor type I (BMPR-I) was immunoprecipitated from the liver lysates of mice fed with regular (control) or ethanol-containing (alcohol) Lieber-De Carli liquid diets, and of mice fed with diets containing 0.2% (normal) or 2% (overload) carbonyl iron, as described in Materials and Methods. The phosphorylation of BMPR-I on serine residues in the immune complexes was determined by western blotting employing an anti-phosphoserine antibody (a), and the total levels of BMPR-I protein were detected by western blotting with an anti-BMPR-I antibody (b). (c) Autoradiographs from different experiments (*n* = 2) were scanned by a densitometer, and phospho-BMPR-I (p-BMPR-I) expression in each sample was quantified by normalizing to immunoprecipitated BMPR-I protein level. Normalized p-BMPR-I expression in alcohol or high iron-fed mice was expressed as fold expression of that in the control mice or in mice fed with normal iron diet.

Bone morphogenetic proteins bind to and signal through type I and type II serine/threonine kinase receptors, BMPR-I and BMPR-II. Upon ligand binding, BMPR-I is phosphorylated. To determine the activation of BMPR-I, we performed immunoprecipitation experiments followed by western blotting, as described in Materials and Methods. BMPR-I immunocomplexes from mouse livers were blotted with an anti-phosphoserine antibody. The level of BMPR-I phosphorylation on serine residues in alcohol-fed mice was not significantly different than that in control mice (Figures 7(a) and 7(c)). However, BMPR-I phosphorylation was induced in the livers of mice fed with high iron diets, which were used as internal controls (Figures 7(a) and 7(c)). We also confirmed by western blotting that equal levels of BMPR-I protein were immunoprecipitated from the livers of alcohol or iron-treated and control mice (Figure 7(b)). Furthermore, control samples, which were immunoprecipitated with normal rabbit IgG also showed no significant BMPR-I phosphorylation (Figure 7(a)). 

**Figure 8:**
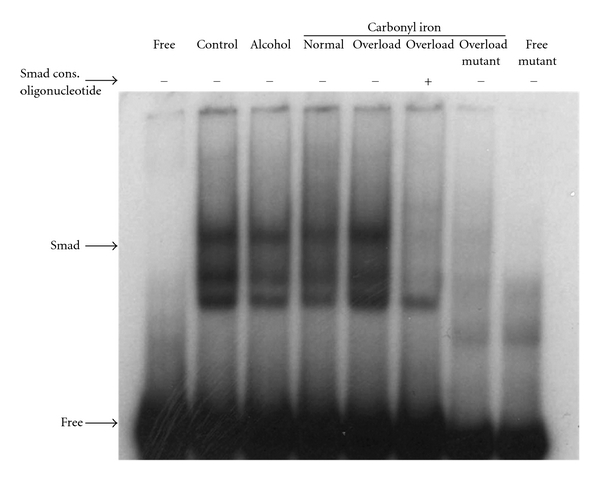
*Smad4 DNA-Binding Activity*. Smad DNA-binding activity was determined by electrophoretic mobility gelshift assays (EMSA), as described in Materials and Methods. 7 *μ*g of nuclear lysate protein isolated from the livers of mice fed with regular (control) or ethanol-containing (alcohol) Lieber-De Carli liquid diets, and of mice fed with diets containing 0.2% (normal) or 2% (overload) carbonyl iron was employed in EMSA. Cold competition with unlabeled Smad consensus (cons.) oligonucleotides and ^32^P-labeled mutant Smad probe were employed to test the specificity of DNA binding, as described in Materials and Methods. The arrows indicate specific Smad DNA complexes and unbound (free) probes. The results are representative of multiple EMSA experiments (*n* = 3).

Smad4 forms a complex with phosphorylated R-Smads and regulates the transcription of target genes. In order to determine the effect of alcohol on Smad4 DNA-binding activity in the liver, we performed electromobility shift assays, as described in Materials and Methods. The DNA-binding activity of Smad4 in liver nuclear lysates from mice with chronic alcohol exposure was not significantly different than that of control mice (Figure 8). However, iron, used as internal control, induced Smad DNA-binding activity (Figure 8). The specificity of DNA-binding activity was also confirmed with both competition tests, using unlabeled (cold) Smad consensus oligonucleotides, and by employing ^32^P-labeled mutant Smad oligonucleotide as a probe in gelshift assays, as described in Materials and Methods (Figure 8). 

**Figure 9:**
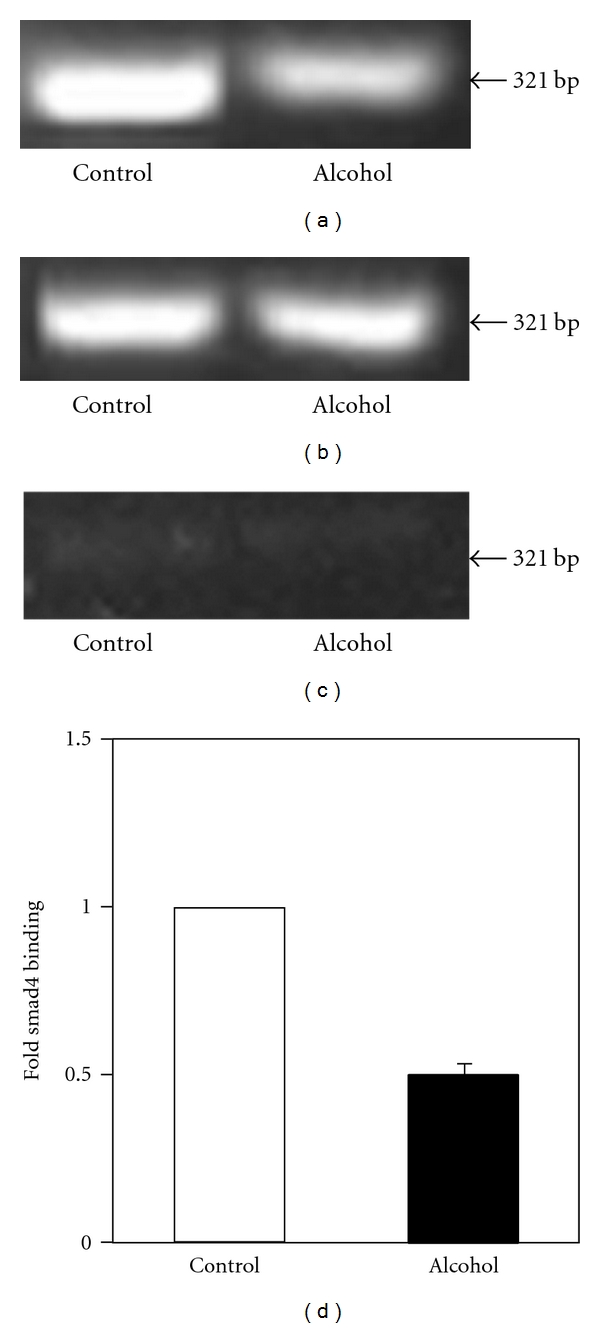
*Smad4 Binding to Mouse Hepcidin Promoter*. Chromatin was isolated from the livers of mice fed with regular (control) or ethanol-containing (alcohol) Lieber-De Carli liquid diets. Chromatin immunoprecipitation was performed with anti-Smad4 antibody (a) or normal rabbit IgG (control) (c). The coimmunoprecipitated DNA and total input DNA (control) (b) were subjected to PCR to amplify a 321 base pair mouse hepcidin promoter region, as described in Materials and Methods. (d) Ethidium bromide-stained agarose gels from different experiments (*n* = 3) were scanned to quantify the amount of Smad4 co-immunoprecipitated DNA by normalizing to input DNA.

In order to determine the effect of chronic alcohol exposure on Smad4-mediated transcription of hepcidin, we performed chromatin immunoprecipitation experiments, as described in Materials and Methods. The binding of Smad4 to hepcidin promoter was significantly attenuated in the livers of mice treated with alcohol compared to control mice (Figures 9(a) and 9(d)). We have also confirmed that the level of total input DNA (see Materials and Methods) was similar in all samples (Figure 9(b)). Furthermore, no significant amplification of hepcidin promoter was observed in chromatin samples, which were immunoprecipitated with the control IgG (Figure 9(c)).

## 4. Discussion

Hepcidin, mainly synthesized in the liver, is the key regulator of iron homeostasis and its expression is also regulated by iron. Alcohol has been shown to suppress hepcidin transcription in the liver leading to elevated iron absorption in the duodenum [9–11, 13]. However, how alcohol attenuates liver hepcidin transcription and function is not completely understood. 

Various signaling mechanisms including BMP-mediated Smad signaling are involved in the regulation of hepcidin transcription in the liver [23–25, 27]. The deletion of Smad4 in the liver also attenuates hepcidin expression and causes pronounced hepatic iron accumulation in mice [32]. BMPs belong to the TGF-*β* superfamily of growth factors. TGF-*β* is one of the main profibrogenic cytokines, which is involved in the progression of alcoholic liver disease [33, 34, 38]. However, the role of BMPs in alcoholic liver disease is unclear. Here, we show that alcohol significantly induces the expression of BMP2 in the liver *in vivo*. Although alcohol is known to alter iron homeostasis [3, 5, 39], unlike iron [25, 27], chronic alcohol exposure did not significantly upregulate the expression of BMP6 in the liver.

Ligand binding induces the phosphorylation of type I BMP receptor (BMPR-I). Upon phosphorylation, BMPR-I stimulates BMP signaling by phosphorylating Smad1, Smad5, and Smad8. Interestingly, despite an alcohol-induced increase in BMP expression, the phosphorylation of Smad1 and Smad5 was not elevated in the livers of mice with chronic alcohol exposure. In contrast, TGF-*β*-mediated phosphorylation of Smad2 was induced by alcohol. BMPR-I receptor is expressed on the plasma membrane. It is feasible that alcohol-mediated inhibition of BMP-mediated Smad signaling may occur in proximity to the cell surface. Our immunoprecipitation studies clearly demonstrate a lack of BMPR-I phosphorylation in the livers of mice with chronic alcohol exposure. The specificity of immune complexes was confirmed by employing control antibodies and IgG light-chain-specific secondary antibodies for western blotting (see Figure 7). Furthermore, we have also confirmed that iron, as an internal control, upregulates the phosphorylation of both BMPR-I, and Smad1 and Smad5 proteins. Of note, we have previously reported that alcohol renders hepcidin insensitive to body iron levels and abolishes its protective role in iron overload [12]. However, whether or not alcohol interferes with iron-mediated activation of hepcidin transcription via BMP/Smad signaling in the liver warrants further investigation. 

The inhibition of BMP receptor activation and signaling by alcohol may involve various mechanisms. Alcohol metabolism is well known to increase the NADH:NAD^+^ ratio and induce hypoxia in the liver. Accordingly, hypoxia has recently been suggested to inhibit hepcidin expression by attenuating Smad signaling in human Huh7 hepatoma cells [40]. Furthermore, hypoxia-induced changes in the NADH : NAD^+^ ratio have been reported to attenuate BMP receptor activation in lung cells [41]. However, it should be noted that alcohol-induced hypoxia is limited to the centrilobular region of the liver [42]. It is therefore possible that other mechanisms besides hypoxia may be involved in alcohol-induced inhibition of BMP-mediated Smad signaling in the liver. For example, changes in inhibitory Smads or a competition between R-Smads activated by TGF-*β* (Smad2, Smad3) and BMPs (Smad1, Smad5, and Smad8). Of note, TGF-*β* receptor has been reported to interact with BMPR-I and inhibit BMP-mediated Smad signaling [43]. Accordingly, we have observed the activation of Smad2, but not of Smad1 or Smad5, in the livers of mice with chronic alcohol exposure. Conversely, recombinant BMP6 has been shown to inhibit TGF-*β*-mediated Smad signaling [44]. Nevertheless, our findings showing alcohol-induced inhibition of Smad DNA-binding activity and the binding of Smad4 to hepcidin promoter strongly suggest that alcohol can directly interfere with nuclear Smad DNA complexes. The regulation of Smad signaling complexes by alcohol may therefore be one of the mechanisms by which alcohol suppresses hepcidin transcription in the liver *in vivo*.

Alcohol metabolism in the liver produces toxic metabolites, such as acetaldehyde and lipid peroxidation products [45, 46]. They, in turn, activate TGF-*β* production and lead to the secretion of extracellular matrix proteins [33]. BMPs have been reported to interact with and antagonize TGF-*β* blocking its profibrogenic activity [47, 48]. By blocking TGF-*β*, BMPs can also modulate cell adhesion and migration [49, 50]. It is therefore possible that the induction of BMP expression in the liver in response to chronic alcohol exposure is associated with antifibrogenic response mechanisms. Furthermore, the inverse effect of alcohol on TGF-*β* and BMP-mediated Smad signaling may be one of the mechanisms involved in the progression of liver fibrosis in alcoholic liver disease. 

The alcohol-induced inhibition of Smad4 binding to hepcidin promoter and suppression of hepcidin transcription in the liver is expected to gradually elevate intestinal iron uptake and iron storage in Kupffer cells. Accordingly, we have previously reported the elevation of hepatic iron levels in rats with chronic alcohol exposure [12]. Iron and alcohol are known to act synergistically to induce liver injury [51–53]. Interestingly, the inhibition of BMP signaling has also been reported in Hfe knockout mice, an animal model for the commonest iron overload disorder, genetic hemochromatosis [54, 55]. This study therefore indicates a role for Smad signaling in the regulation of iron metabolism by alcohol, which may have implications for alcoholic liver disease and also genetic hemochromatosis in conjunction with alcohol.

## 5. Conclusions

Bone morphogenetic protein signaling has recently been shown to induce hepcidin transcription in the liver. BMP and TGF-*β* both belong to the same family of growth factors and stimulate the Smad signaling pathway. Alcohol is known to induce TGF-*β* expression, which plays a role in liver fibrinogenesis, whereas the effect of alcohol on BMP signaling is unknown. Here, we show that similar to TGF-*β*, BMP protein expression was also upregulated in the liver. However, alcohol exerted different effects on TGF-*β*-mediated Smad2 activation and BMP-mediated Smad1 and Smad5 activation. The inhibitory effect of alcohol on BMP-mediated Smad signaling may occur in proximity to the cell surface by interfering with the activation of BMP receptor type I. This subsequently resulted in the inhibition of Smad4 binding to hepcidin promoter in the livers of mice with chronic alcohol exposure. Collectively, these findings strongly suggest that the simultaneous inhibition of BMP-mediated Smad activation and stimulation of TGF-*β*-mediated Smad activation by alcohol may be involved in the suppression of liver hepcidin transcription and deregulation of iron metabolism by alcohol *in vivo*. Iron and alcohol act synergistically to induce liver injury. Further understanding of the role of alcohol in Smad signaling and hepcidin transcription will help to elucidate the mechanisms of liver injury observed in patients with alcoholic liver disease or with genetic hemochromatosis and alcohol abuse.

## Figures and Tables

**Table 1:** Mouse-specific sequences of real-time quantitative PCR probe and primers.

Gene	Forward primer (5^'^-3^'^)	Reverse primer (5^'^-3^'^)	Taqman probe (5^'^-3^'^)
BMP2	GCATCCAGCCGACCCTT	GCCTCAACTCAAATTCGCTGA	TCCCGGCCTTCGGAAGACGTC
BMP4	GGACTTCGAGGCGACACTTC	TTGCTAGGCTGCGGACG	ACAGATGTTTGGGCTGCGCCG
BMP6	CCTCTTCTTCGGGCTTCCTC	CCTTTTGCATCTCCCGCTT	ATCGGCGGCTCAAGACCCACG
Hepcidin	ACTCGGACCCAGGCTGC	AGATAGGTGGTGCTGCTCAGG	TGTCTCCTGCTTCTCCTCCTTGCCA
